# Correction: Pre-post synaptic alignment through neuroligin-1 tunes synaptic transmission efficiency

**DOI:** 10.7554/eLife.65689

**Published:** 2020-12-16

**Authors:** Kalina T Haas, Benjamin Compans, Mathieu Letellier, Thomas M Bartol, Dolors Grillo-Bosch, Terrence J Sejnowski, Matthieu Sainlos, Daniel Choquet, Olivier Thoumine, Eric Hosy

Haas KT, Compans B, Letellier M, Bartol TM, Grillo-Bosch D, Sejnowski TJ, Sainlos M, Choquet D, Thoumine O, Hosy E. 2018. Pre-post synaptic alignment through neuroligin-1 tunes synaptic transmission efficiency. *eLife* **7**:e31755. doi: 10.7554/eLife.31755.

Published 25, July 2018

In the published article, Figure 3A inadvertently presented the wrong control inset. The corrected Figure 3A is now shown. No further changes were made to the text and figure legend. Please note that this correction does not affect the results and conclusions of the original paper.

The corrected Figure 3A is shown here:

**Figure 1:**
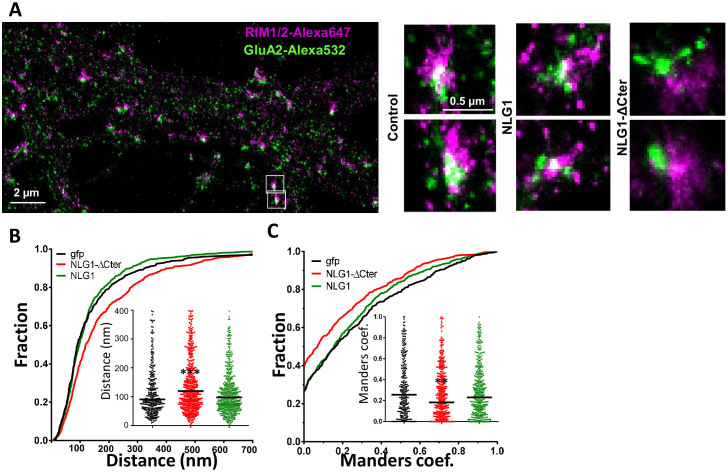


The originally published Figure 3A is also shown for reference:

**Figure 2:**
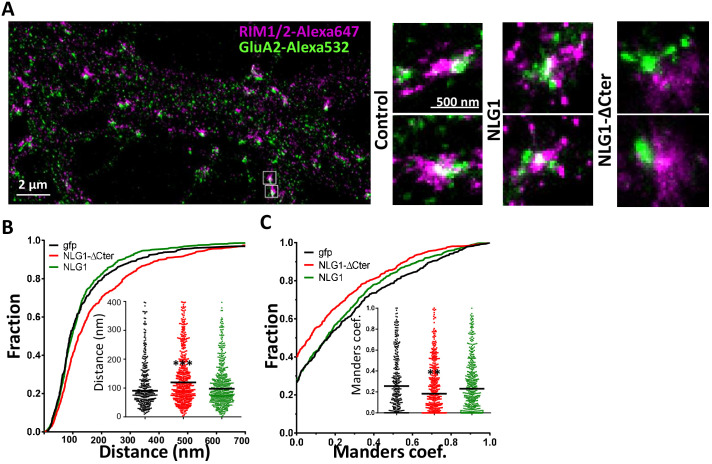


The article has been corrected accordingly.

