# Supplementary material for: Pre-post synaptic alignment through neuroligin-1 tunes synaptic transmission efficiency
Source: eLife. 2018 Jul 25;7:e31755. doi: 10.7554/eLife.31755 (PMC6070337; doi:10.7554/eLife.31755)
Supplement: Transparent reporting form [file elife-31755-transrepform.docx]

***eLife’s* transparent reporting form**

We encourage authors to provide detailed information *within their submission* to facilitate the interpretation and replication of experiments. Authors can upload supporting documentation to indicate the use of appropriate reporting guidelines for health-related research (see [EQUATOR Network](http://www.equator-network.org/%20)), life science research (see the [BioSharing Information Resource](https://biosharing.org/)), or the [ARRIVE guidelines](http://www.plosbiology.org/article/info:doi/10.1371/journal.pbio.1000412) for reporting work involving animal research. Where applicable, authors should refer to any relevant reporting standards documents in this form.

If you have any questions, please consult our Journal Policies and/or contact us: [editorial@elifesciences.org](mailto:editorial@elifesciences.org).

**Sample-size estimation**

- You should state whether an appropriate sample size was computed when the study was being designed
- You should state the statistical method of sample size computation and any required assumptions
- If no explicit power analysis was used, you should describe how you decided what sample (replicate) size (number) to use

Please outline where this information can be found within the submission (e.g., sections or figure legends), or explain why this information doesn’t apply to your submission:

No sample size calculation was performed. However, sample size number was based on values currently used in the literature (e.g. 10-15 cells per condition for electrophysiological recordings, 15-30 cells per condition for immunocytochemistry and live experiments).

Based on the statistical tests perfomed, this sample size was appropriate to establish significant differences between groups.

**Replicates**

- You should report how often each experiment was performed
- You should include a definition of biological versus technical replication
- The data obtained should be provided and sufficient information should be provided to indicate the number of independent biological and/or technical replicates
- If you encountered any outliers, you should describe how these were handled
- Criteria for exclusion/inclusion of data should be clearly stated
- High-throughput sequence data should be uploaded before submission, with a private link for reviewers provided (these are available from both GEO and ArrayExpress)

Please outline where this information can be found within the submission (e.g., sections or figure legends), or explain why this information doesn’t apply to your submission:

Figure1: between 9 to 12 individuals cells from 3 independent dissections; between 200 to 500 synapses

Figure 2: between 300 to 500 co-localized objects, issue of 10 dual color acquired cells, 3 independent dissections

Figure 3: between 350 to 600 co-localized objects, issue of 12 dual color acquired cells, 3 independent dissections

Figure 4: 14, 14 and 15 cells has been used for miniature analysis on cell culture (representing more than 5000 individual synaptic currents), 3 independent dissections; for paired-patch brain slices experiments: minimum of 6 paired cells, at least independent dissections

Figure 5: between 400 to 1000 co-localized objects, issue of 9 dual color acquired cells, 3 independent dissections; and at least 9 cells for electrophysiology experiments

No outlayer has been suppressed

All important n values are reported either in the results or in the figure legends

**Statistical reporting**

- Statistical analysis methods should be described and justified
- Raw data should be presented in figures whenever informative to do so (typically when N per group is less than 10)
- For each experiment, you should identify the statistical tests used, exact values of N, definitions of center, methods of multiple test correction, and dispersion and precision measures (e.g., mean, median, SD, SEM, confidence intervals; and, for the major substantive results, a measure of effect size (e.g., Pearson's r, Cohen's d)
- Report exact p-values wherever possible alongside the summary statistics and 95% confidence intervals. These should be reported for all key questions and not only when the p-value is less than 0.05.

Please outline where this information can be found within the submission (e.g., sections or figure legends), or explain why this information doesn’t apply to your submission:

Figure1: B: one way anova: p=0.0004; dunnett’s multicomparison test: p=0.0299; p=0.0002

C: one way anova: p<0.0001; dunnett’s multicomparison test: p<0.0001; p=0.7268

D: one way anova: p<0.0001; dunnett’s multicomparison test: p<0.0001; p=0.9809

E: one way anova: p=0.0063; dunnett’s multicomparison test: p<0.0001; p=0.3206

Figure3: B: one way anova: p=0.0002 Brown-Forsythe test

C: one way anova: p=0.0015 Brown-Forsythe test

Figure4: C: one way anova: p=0.0006; dunnett’s multicomparison test: p=0.0174; p=0.2596

H: paired t test, p=0.9935

I: paired t test, p=0.0186

Figure5: B: one way anova: p<0.0001 Brown-Forsythe test

C: one way anova: p<0.0001 Brown-Forsythe test

D: one way anova: p=0.0046 dunnett’s multicomparison test: p0.0055; p=0.6801

E: one way anova: p=0.7547

Statistical tests are justified in the methods section, n and p values are reported in the results part or in the figure legends

(For large datasets, or papers with a very large number of statistical tests, you may upload a single table file with tests, Ns, etc., with reference to sections in the manuscript.)

**Group allocation**

- Indicate how samples were allocated into experimental groups (in the case of clinical studies, please specify allocation to treatment method); if randomization was used, please also state if restricted randomization was applied
- Indicate if masking was used during group allocation, data collection and/or data analysis

Please outline where this information can be found within the submission (e.g., sections or figure legends), or explain why this information doesn’t apply to your submission:

All experiments from acquisition to analysis have been done blind, allocation are realized after analysis

**Additional data files (“source data”)**

- We encourage you to upload relevant additional data files, such as numerical data that are represented as a graph in a figure, or as a summary table
- Where provided, these should be in the most useful format, and they can be uploaded as “Source data” files linked to a main figure or table
- Include model definition files including the full list of parameters used
- Include code used for data analysis (e.g., R, MatLab)
- Avoid stating that data files are “available upon request”

Please indicate the figures or tables for which source data files have been provided:

Matlab script for dual color imaging analysis is available upon request
